# Improved Early Postresuscitation EEG Activity for Animals Treated with Hypothermia Predicted 96 hr Neurological Outcome and Survival in a Rat Model of Cardiac Arrest

**DOI:** 10.1155/2013/312137

**Published:** 2013-12-04

**Authors:** Bihua Chen, Feng-Qing Song, Lei-Lei Sun, Ling-Yan Lei, Wei-Ni Gan, Meng-Hua Chen, Yongqin Li

**Affiliations:** ^1^School of Biomedical Engineering, Third Military Medical University and Chongqing University, Chongqing 400038, China; ^2^Institute of Cardiovascular Diseases, The First Affiliated Hospital of Guangxi Medical University, Nanning 530021, China

## Abstract

*Purpose.* To investigate the effect of hypothermia on 96 hr neurological outcome and survival by quantitatively characterizing early postresuscitation EEG in a rat model of cardiac arrest. *Materials and Methods.* In twenty male Sprague-Dawley rats, cardiac arrest was induced through high frequency transesophageal cardiac pacing. Cardiopulmonary resuscitation was initiated after 5 mins untreated arrest. Immediately after resuscitation, animals were randomized to either 2 hrs of hypothermia (*N* = 10) or normothermia (*N* = 10). EEG, ECG, aortic pressure, and core temperature were continuously recorded for 6 hrs. Neurological outcome was evaluated daily during the 96 hrs postresuscitation period. *Results.* No differences in the baseline measurements and resuscitation outcome were observed between groups. However, 96 hr neurological deficit score (204 ± 255 versus 500 ± 0, *P* = 0.005) and survival (6/10 versus 0/10, *P* = 0.011) were significantly better in the hypothermic group. Quantitative analysis of early postresuscitation EEG revealed that burst frequency and spectrum entropy were greatly improved in the hypothermic group and correlated with 96 hr neurological outcome and survival. *Conclusion.* The improved burst frequency during burst suppression period and preserved spectrum entropy after restoration of continuous background EEG activity for animals treated with hypothermia predicted favorable neurological outcome and survival in this rat model of cardiac arrest.

## 1. Introduction

Out-of-hospital cardiac arrest (CA) is a major public health problem all over the world. Each year, an estimated 325,000 victims in USA, 350,000 in Europe, and 544,000 in China suffer out-of-hospital CA [1–3]. Despite efforts to improve outcomes from CA, the overall survival is less than 10% among patients successfully resuscitated [4, 5]. In patients who achieved return of spontaneous circulation (ROSC), the resulting anoxic ischemia brain injury is a major cause of morbidity and mortality [6, 7]. The greatest postresuscitation emphasis has mainly been on preserving neurologic function [8].

Among all postresuscitation care suggested and/or recommended, therapeutic hypothermia (TH) is the most persuasive intervention that can significantly improve neurologic recovery and survival after resuscitation from CA [9, 10]. However, patient selection and the optimization of postarrest hypothermia treatment remain problematic issues because there are no clinically validated tools to determine who might benefit from the therapy, how long hypothermia should be conducted, and how to avoid/reduce occurrence of complications [9, 10]. Early prediction of outcome may be, in fact, an important aspect to be considered during the postresuscitation care in order to avoid the likelihood of unnecessary prolongation of TH when a good functional recovery has already been achieved or to avoid unjustified withdrawal of care if the protection has not been fully achieved yet. For years, neurological examination and electrophysiological studies have guided physicians in predicting outcome in comatose survivors of CA, including pupillary light response, serum neuron-specific enolase, somatosensory evoked potentials, and combinations thereof [11–14]. But early prognostication remains challenging, especially because the predictive values of clinical, biochemical, and electrophysiological variables have become uncertain after the introduction of TH [15–17].

The electroencephalogram (EEG), which reflects part of the function of cortical neurons, is very sensitive to ischemia. Previous studies found that EEG burst characteristics were associated with neurological recovery in animal model of CA from asphyxia [18, 19]. At the same time, observational clinical studies reported that persistence of isoelectric activity, burst suppression, or generalized epileptiform discharges on EEG was associated with poor outcomes [20–23]. Although unprocessed EEG interpretation observed during the early stage after resuscitation has been used to assist the prediction of a poor outcome in comatose survivors without hypothermia with some success, the prognostic accuracy was insufficient, especially in the era of hypothermia [24–27]. Meanwhile, the EEG literatures of clinical study are confounded by different classification systems, causes of CA, arrest time, duration of cardiopulmonary resuscitation (CPR), medications used, and intervals of recordings after resuscitation [28]. The characteristics of EEG during early postresuscitation period and the effect of hypothermia on EEG recovery and its prognostication value are still unclear [9, 10, 28].

In the present study, we investigated the effect of mild hypothermia on EEG recovery, as well as the relationship between characteristics of early postresuscitation EEG activities and 96 hr neurological outcome and survival in a rat model of CA.

## 2. Materials and Methods

This study was approved by the ethics of animal investigation committee of Guangxi Medical University. All animals received humane care in compliance with the Principles of Laboratory Animal Care and Guide for the Care and Use of Laboratory Animals [29].

### 2.1. Animal Preparation

Twenty male Sprague-Dawley rats weighing 230 to 334 g were fasted overnight but had free access to water. Anesthesia was initiated by intramuscular injection of (0.3 g/kg) chloral hydrate. Additional doses of 0.03 g/kg were administered at intervals of 1 hr or when required to maintain anesthesia, except when no anesthetic agents were administrated for 30 mins before induction CA. The trachea was orally intubated with a 14-gauge cannula for mechanical ventilation by a volume-controlled ventilator (ALC-V9, Alcott Biotech CO., Shanghai, China) at tidal volume of 6 mL/kg. A polyethylene tubing PE50 (Instech Laboratories Inc. Plymouth Meeting, PA, USA) was advanced from the left femoral artery into the thoracic/descending aorta for measurement of arterial pressure. Through the right external jugular vein, another PE50 catheter was advanced into the right atrium for measurement of right atrial pressure and for the administration of chloral hydrate. Aortic and right atrial pressures were measured with two high-sensitivity transducers via a multiparameter patient monitor (Datascope 3000, Datascope Corp. Paramus, NJ, USA). A thermocouple microprobe (IT-21, Physitemp Instruments, Clifton, NJ, USA) was inserted into the right femoral artery and advanced to the descending aorta for measurement of blood temperature. A 5F pacing electrode with two 1 mm ring electrodes and an interelectrode distance of 5 mm was inserted orally into the esophagus of the rats about 7 cm in depth for inducing ventricular fibrillation (VF). All of the catheters were flushed intermittently with saline containing 5 IU/mL of crystalline bovine heparin.

### 2.2. Experimental Procedures

After collection of baseline data, VF was induced through high frequency transesophageal cardiac pacing with an alternating voltage of 24 V as previously described [30]. The stimulation was continued for 1 min to prevent spontaneous cardiac reversion. Mechanical ventilation was discontinued when cardiac pacing was started. After 5 mins of untreated CA, CPR, including manual chest compression and mechanical ventilation with air, was begun. Chest compression was performed at a rate of 200 compressions per minute, with a depth of 25%–30% of the anterior posterior diameter of the animal's chest and with equal compression-relaxation duration by the same investigator. After 1 min of CPR, one dose of epinephrine (20 *μ*g/kg) was given through the right atrial catheter. An organized cardiac rhythm with mean aortic pressure of >60 mmHg for a minimum of 5 mins was defined as successful ROSC. CPR was continued unless the animal was either successfully resuscitated or pronounced dead after a total of 15 mins CPR.

Immediately after resuscitation, animals were randomized to hypothermic or normothermic group and monitored in an intensive care setting for additional 6 hrs. For animals assigned to TH, surface cooling was induced with the aid of ice packs and an electrical fan. Once the target temperature reached 33.5°C, it was maintained over the first 2 hr of postresuscitation and then gradually returned to 37.0°C over a rewarming period of 2 hrs. For those animals subjected to normothermic control, blood temperature was maintained at 37.0 ± 0.3°C during the 6 hrs postresuscitation observational period. All catheters, including the temperature transducer and endotracheal tube, were then removed and wounds were surgically sutured. Animals were then returned to their cages and observed for 96 hrs.

The neurological functions were assessed daily during the 96 hr postresuscitation period according to neurologic deficit scores (NDS), which was developed to evaluate neurological outcome after global cerebral ischemia for rats [31]. Details of NDS scales are illustrated in Table 1. The total score ranges from 0 to 500, representing no observational neurological deficit and brain death.

### 2.3. Measurements

The ECG, EEG, pressure measurements, and core temperature were continuously measured and recorded through a data acquisition system supported by WinDaq hardware/software (DATAQ Instruments Inc., Akron, OH, USA) at a sample rate of 300 Hz. Four subdermal needle electrodes (right-frontal, right-parietal, left-frontal, and left-parietal) placed over the surface of the skull were used for bipolar EEG measurement and recording. A two-channel EEG differential preamplifier (PRE-ISO.EEG100, Xiangyun Computing Technology, Beijing, China) was used for signal amplification and condition. The amplifier gain of each channel was set at 10,000 and the cutoff frequencies were set at 0.3 and 70 Hz for the high-pass and low-pass filters, respectively.

EEG analysis was performed offline after the experiment was concluded. All of the EEG patterns were visually annotated by an investigator and were further confirmed by another medical doctor who was blinded to the outcome. During the 6 hrs observational period, the EEG pattern was classified as one of the three following categories [25, 32]: isoelectric/suppression, burst suppression, and continuous background EEG activity. Isoelectric/suppression was defined as total absence of any visible EEG activity during a 60 secs recording episode. Burst suppression was defined by the presence of clear increases in amplitude (bursting) followed by interburst intervals of at least 0.5 sec without EEG activity or low amplitude activity (less than 10 *µ*V). Bursts were required to have EEG amplitude >10 *µ*V in both left and right channels. Characteristics of earlier postresuscitation EEG, including the onset time of identifiable EEG burst, the frequency of bursts during the burst suppression period, the time of recovery of continuous background EEG activity, and the spectral entropy (SE) of continuous background EEG [33], were quantitatively analyzed.

SE was calculated using the Welch averaged periodogram method from consecutive nonoverlapping epochs of 60 seconds by MATLAB 7.0 (The MathWorks, Inc., Natick, MA, USA). Linear detrending and Hanning windowing were applied to the signal before applying the Fast Fourier Transform. The sum of the magnitudes (EEG power in different subbands) in each individual predetermined frequency band that represents Delta (0.5–4 Hz), Theta (4–8 Hz), Alpha (8–13 Hz), and Beta (13–30 Hz) waves was calculated and the probability density function of each wave band was then computed as

(1) $$\begin{array}{c} p^{i}=\frac{X_{i}}{\sum_{i=1}^{N}X_{i}}, \end{array}$$

where *X*_*i*_ represents the total energy of the *i*th band, *p* ^*i*^ is the probability mass function of the spectrum in each band, and *N* is the total number of bands. The SE was calculated as

(2) $$\begin{array}{c} \text{SE}=-\frac{\sum_{i=1}^{N}p^{i}*\log_{2}p^{i}}{\log_{2}N}. \end{array}$$

### 2.4. Statistical Analysis

Data were presented as Mean ± SD. The 6 hrs EEG analysis and 96 hrs neurologic outcome and survival served as primary variables between experimental groups. For baseline and experimental measurements between groups, two-tailed Student's *t*-test was used. Quantitative EEG characteristics were analyzed by two-way analysis of variance (ANOVA) for post hoc comparison between the two experimental groups. Kaplan-Meier analysis and the log-rank test were used to calculate survival rates. The associations between characteristic indices of EEG and 96 hr neurologic outcome and survival were analyzed using Spearman's correlation and logistic regression. A *P* < 0.05 was regarded as statistically significant.

## 3. Results and Discussions

### 3.1. Results

The detailed baseline and experimental measurements are presented in Table 2. There were no differences in body weight and baseline measurements of heart rate, body temperature, and mean arterial pressure between groups.

**Figure 1:**
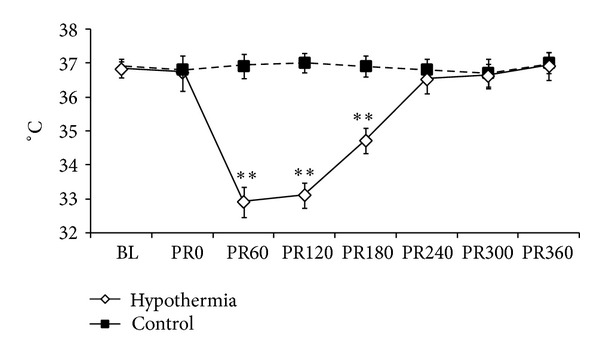
Core temperature before and after resuscitation. PR: postresuscitation. **P < 0.01 compared with normothermic control.

CA was successfully induced in all animals after 1 min of transesophageal cardiac pacing. The cardiac rhythm rapidly deteriorated from VF to pulseless electrical activity (PEA) before CPR was initiated. All of the twenty rats were successfully resuscitated without the aid of defibrillatory shocks and survived to 6 hrs. No differences in the duration of CPR time (93.3 ± 19.6 versus 86.1 ± 11.9 secs, *P* = 0.34) and coronary perfusion pressure during CPR (21.6 ± 3.7 versus 21.2 ± 3.5 mmHg, *P* = 0.78) were observed between groups.  Figure 1 shows the core temperature measured during the experiment. For control group, the body temperature was maintained between 36.6°C and 37.4°C during the 6 hrs observational period. For hypothermic group, the target core temperature was obtained within 26.9 mins (15.7 ± 5.0 mins) and maintained for 2 hrs.

**Figure 2:**
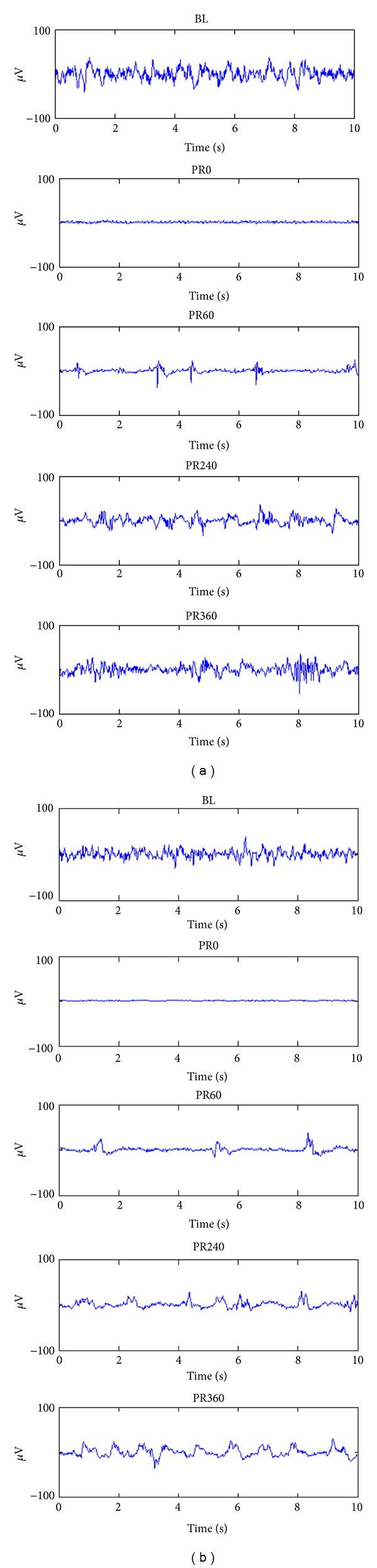
Examples of the evolution of EEG patterns for normothermia (a) and hypothermia (b). BL: baseline. PR: postresuscitation.

**Figure 3:**
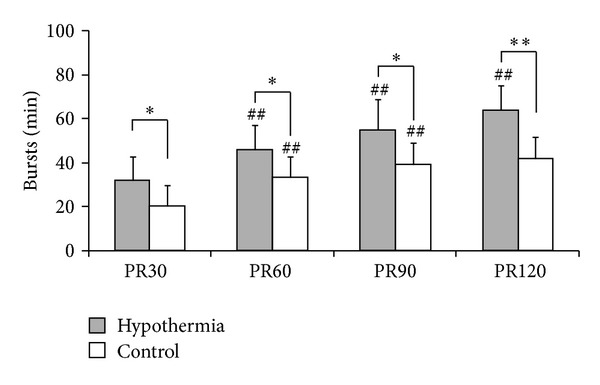
Burst frequency during the first 2 hr postresuscitation (PR) burst suppression period. ^##^ *P* < 0.01 compared with previous measurement. ^#^ *P* < 0.05 compared with previous measurement. **P < 0.01 compared with normothermic control. *P < 0.05 compared with normothermic control.

As illustrated in Figure 2, all of the animals showed the same EEG recovery pattern during the 6 hrs EEG recording period in the order of isoelectric tracing, burst suppression, and continuous background EEG activity. However, the onset time of identifiable EEG burst (15.1 ± 1.9 versus 21.5 ± 6.0 mins, *P* = 0.008) and the time of recovery of continuous background EEG activity (171.2 ± 15.2 versus 239.5 ± 38.4 mins, *P* = 0.0002) were significantly shorter in the hypothermic group compared to the normothermic one. For rats treated with hypothermia, the frequency of burst was continuously increasing during the first 2 hrs after resuscitation. For normothermic rats, burst frequency was also increasing during the first 90 mins but this trend was not persisted at later burst suppression period. The burst frequency was significantly higher in the hypothermic group compared with that in control (Figure 3).

**Figure 4:**
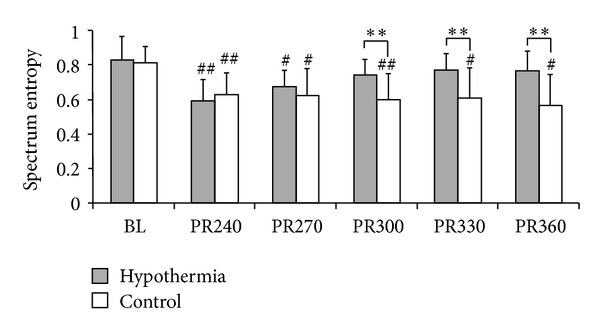
Spectrum entropy measurement of EEG. ^##^ *P* < 0.01 compared with baseline. ^#^ *P* < 0.05 compared with baseline. **P < 0.01 compared with control. BL: baseline. PR: postresuscitation.

Since no difference in SE measurements was observed between the two EEG channels during the observational period, data were reported by the average of left and right channels. The baseline and postresuscitation SE measurements of continuous background EEG are reported in Figure 4. There were no differences in baseline measurements between groups (0.829 ± 0.133 versus 0.811 ± 0.096, *P* = 0.740). Four hrs after ROSC, the EEG evolved to continuous background activity in all of the hypothermic animals, but 5 of the normothermic animals were still on the stage of burst suppression pattern and eventually evolved to continuous background EEG activity within an additional 1 hr. Five hrs after ROSC, SE was restored to baseline in the hypothermic animals (0.741 ± 0.088 versus 0.829 ± 0.133, *P* = 0.080) and was significantly improved compared with that in normothermic ones (0.741 ± 0.088 versus 0.597 ± 0.146, *P* = 0.018), whereas a significant reduction in the control group was observed compared with baseline (0.597 ± 0.146 versus 0.811 ± 0.096, *P* = 0.003). This trend persisted to the end of the 6 hrs recording period (Figure 4) and the SE was significantly higher for hypothermic rats compared with normothermic control (0.776 ± 0.112 versus 0.563 ± 0.179, *P* = 0.009).

**Figure 5:**
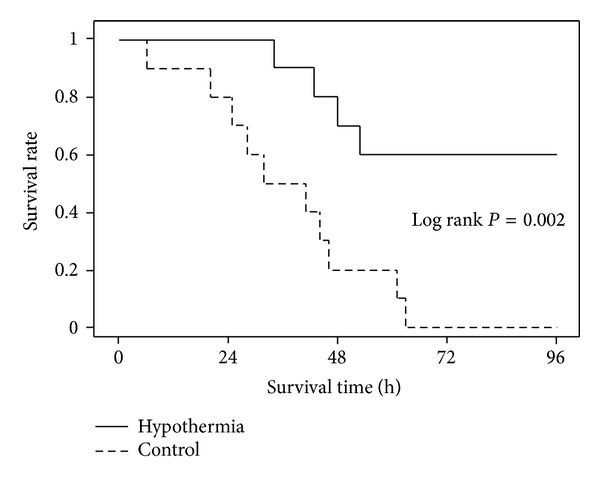
Kaplan-Meier analysis of cumulative survival at 96 hrs postresuscitation.

The neurological outcome measured by NDS was significantly better in the hypothermic animals compared with that in control during the 96 hrs postresuscitation period (Table 3). As shown in the survival curve (Figure 5), all of the hypothermic animals survived to 24 hrs and 6 of them survived to 96 hrs. On the contrary, 8 of the normothermic animals survived to 24 hrs and none survived to 72 hrs.

**Figure 6:**
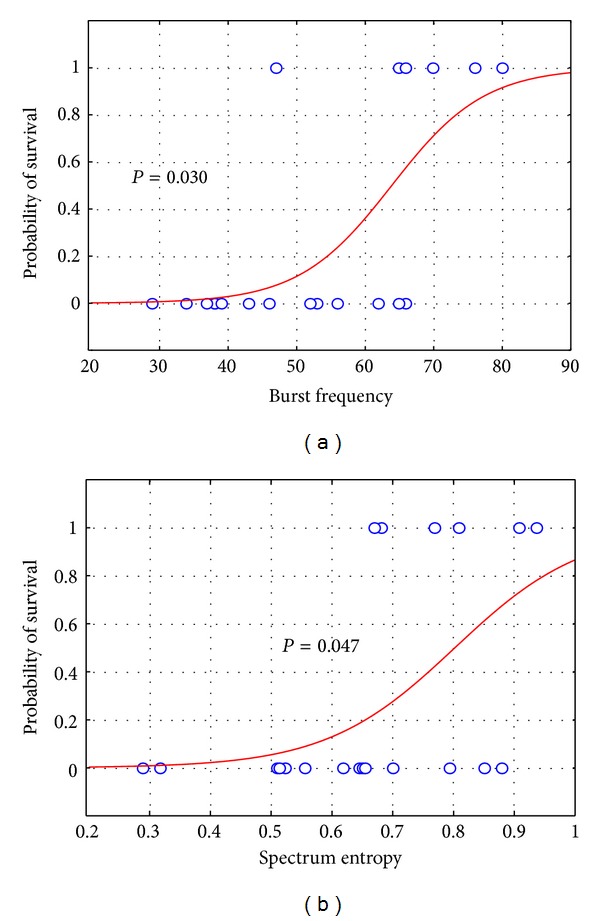
Logistic regression analysis of probability of survival.

The correlation analysis showed that the onset time of EEG bursting (*r* = 0.532, *P* = 0.016), burst frequency at 2 hr (*r* = −0.685, *P* = 0.001), the time of recovery of continuous background EEG activity (*r* = 0.692, *P* = 0.001), and 6 hr SE (*r* = −0.501, *P* = 0.024) were correlated with 96 hr neurological outcome. Single logistic regression analysis (Figure 6) indicated that burst frequency at 2 hr postresuscitation (*P* = 0.030) and SE at 6 hr postresuscitation (*P* = 0.047) were independently predictive of 96 hr survival.

### 3.2. Discussion

This study demonstrated that mild hypothermia improved the recovery of earlier postresuscitation EEG by shortening the isoelectric period, increasing the burst frequency, accelerating the restoration of continuous background EEG activity, and enhancing the irregularity of brain rhythm in a rat model of CA. The results indicated that quantitative EEG characteristics of earlier postresuscitation EEG activity, including improved burst frequency during hypothermia and preserved SE during normothermia, correlated with better neurologic recovery and independently predicted 96 hr survival.

Ischemic brain injury affects synaptic transmission, axonal conduction, and cellular action potential firing in a sequential manner and plays a critical role in determining characteristics of EEG [20]. Since the EEG provides an insight into the thalamocortical function and has been used for prognostication after resuscitation from CA during normothermia, the development of accurate monitoring techniques employing EEG to evaluate the effectiveness of hypothermia and early prediction of neurological outcome may be anticipated [24]. Earlier studies that investigated the effects of changes in brain temperature on EEG showed that hypothermia had a similar influence on EEG in animals and humans [34]. In animal models of asphyxia, hypothermia has been demonstrated to improve EEG restoration after reperfusion by increasing the burst frequency during the early postresuscitation period [35–37]. But in another study investigating the early EEG recovery with temperature manipulation after CA in rats, Jia et al. [38] reported that burst frequency correlated strongly with 72 hr NDS in normothermic rats but not in hypothermic or hyperthermic rats. In attempt to determine a prognostic indicator, quantitative EEG analysis including cepstral distance, EEG entropy, and information quantity has been applied in animal studies [37–39]. Although these measurements were proved to be associated with neurological recovery, the prognostication for survival has not been demonstrated in these studies. Moreover, the animal model of CA induced from asphyxia was more gradual and caused different morphologic patterns of brain damage in contrast to the sudden onset of VF, which was the predominant cause of CA in out-of-hospital adults [40, 41]. Effects of temperature manipulation on EEG and its prognostic ability have also been studied in patients treated with hypothermia, in whom standard EEG was performed after they were successfully resuscitated from CA. Rundgren et al. [25, 26] found that a continuous EEG pattern at the time of normothermia was discriminative for regaining consciousness for hypothermia-treated CA survivors. Wennervirta et al. [42] demonstrated that quantitative EEG variables, including burst suppression ratio, response entropy, state entropy, and wavelet subband entropy differed between good and poor outcome groups in hypothermia-treated patients. Rossetti et al. [16] showed that hypothermia might modify the outcome prediction after CA: an unreactive EEG background was incompatible with good long-term neurological recovery but strongly associated with in-hospital mortality. Leary et al. [43] reported that bispectral index (BIS) values of EEG at 24 hr postresuscitation were correlated with neurological outcomes in patients who underwent hypothermia treatment. But BIS was insufficient to predict good neurologic survival. Cloostermans et al. [27] proved that continuous EEG patterns within 12 hrs predicted good outcome while an isoelectric or low-voltage electroencephalograms after 24 hrs predicted poor outcome in patients treated with hypothermia. But the sensitivity for prediction of good outcome was low (43%). Oh et al. [44] confirmed that a continuous normal voltage EEG activity immediately after ROSC predicted good outcome, with a sensitivity of 57% and specificity of 96%. All of these studies suggested the need for continuous EEG monitoring in patients treated with hypothermia to aid in prognosis and guide management.

In our study, the recovery of EEG activity was consistent with earlier animal studies. But the isoelectric and burst suppression period were significantly shorter and the bursting frequency was significantly higher in the animals treated with hypothermia that had a good neurological outcome compared to those that were normothermic and had a poor neurological recovery. Furthermore, both the onset time of EEG bursting and the time of recovery of continuous background EEG activity were correlated with 96 hr neurological outcome. The neuroprotection effects of hypothermia therefore could be reflected by the improvements in the characteristics of early postresuscitation EEG activity, including shortening the isoelectric period, accelerating the restoration of continuous background EEG, and the increasing the frequency of burst.

Even though characteristics of burst provided important prognostic information after treated with hypothermia, but morphological pattern of EEG activity might not be entirely a marker of good/poor neurological outcome. In our study, EEG was evolved from burst suppression to continuous background activity within 5 hrs in both hypothermic and normothermic groups. This was controversial with previous reports that appearing of continuous background EEG activity was associated with good outcome [25–27, 42, 44]. To quantitatively characterize EEG waveform when continuous background activity was restored, the SE, which provides a quantitative measure of the degree of disorder in brain injury and recovery, was analyzed [45]. For animals treated with hypothermia, the SE was restored to baseline at 5 hr postresuscitation and had significantly higher values in contrast to normothermic animals. The preserved SE after the restoration of continuous background EEG activity was associated with good neurological outcome and predictive survival. C characteristics of early postresuscitation EEG activity at different stages therefore provided indicative information of hypothermic management, especially for those patients who still had a poor neurological prognostication after hypothermia therapy. The potential clinical application of this result is that severely abnormal EEG during earlier postresuscitation period with high probability of poor outcome may indicate the need for hypothermia, while EEG remains discontinuous or continuous EEG background with low probability of good outcome after rewarming may suggest a severe brain injury and the requirement for deeper/longer hypothermia or other postresuscitation cares.

There are several limitations to be considered in the current study. First, although a rat model of VF was used in this study, VF evolved to PEA after successful induction of CA in all animals and no defibrillation shock was needed to resuscitate the animals. Therefore the effect of TH on EEG activity and its prognostic value for CA that was treated with defibrillatory shocks still need to be investigated. Secondly, our study suggested that characteristics of burst suppression and preserved SE may serve as predictors of favourable neurologic outcome after CA in rats treated with hypothermia, but effects of delayed hypothermia or different cooling methods on EEG recovery have not been evaluated. Thirdly, although EEG analysis may provide useful information of neurologic recovery during TH, whether EEG measurement can be used to guide hypothermia therapy is still uncertain. Therefore, a combination of EEG and other methods such as heart rate variability analysis together with biochemical markers may improve the prognostication capability.

## 4. Conclusion

The present study suggests that mild hypothermia greatly improved EEG recovery after resuscitation. Improved burst frequency and preserved SE for animals treated with hypothermia were associated with better neurological outcome and predicted 96 hr survival in this rat model of CA.

## Figures and Tables

**Table 1:** Neurological deficit score (NDS) scale.

Category	Item	Score
Level of consciousness (spontaneous attention to environment and reaction to pinching of ear or tail)	Good attention and brisk response	0
	Sluggish response and no attention	50
	No response	100

Respiration (breathing frequency)	Normal or higher (over 80/min)	0
	Decreased	50
	Apnea	100

Cornea reflex (touch center of cornea with hemostat)	Brisk	0
	Sluggish	20
	Absent	40

Cranial reflex or gag reflex (stimulation with catheter)	Brisk	0
	Sluggish	15
	Absent	30

Auditory reflex (bang metal cop with clamp)	Brisk	0
	Sluggish	15
	Absent	30

Motor sensory function (righting reflex)	Turn spontaneously	0
	Sluggish, partly	50
	No turning attempts	100

Behavior (spontaneous or stimulated)	Moving body, forward movements walking	0
	Movements of the head, looking around	50
	No movements except breathing or not at all	100

**Table 2:** Baseline and experimental measurements.

Measurements	Hypothermia (*N* = 10)	Control (*N* = 10)	*P *value
Body weight, (g)	281.9 ± 34.5	295.8 ± 21.4	0.30
Heart rate, (beats/min)	389.8 ± 53.2	385.1 ± 44.0	0.83
Baseline temperature, (°C)	36.8 ± 0.2	36.9 ± 0.2	0.54
Mean arterial pressure, (mmHg)	109.9 ± 11.5	104.4 ± 16.3	0.40
Cardiopulmonary resuscitation time, (secs)	93.3 ± 19.6	86.1 ± 11.9	0.34
Coronary perfusion pressure, (mmHg)	21.6 ± 3.7	21.2 ± 3.5	0.78
Total chloride hydrate volume, (mL)	1.1 ± 0.2	0.9 ± 0.1	0.07

**Table 3:** Neurological deficit score (NDS) and survival.

Outcome	24 hr	48 hr	72 hr	96 hr
NDS				
Hypothermia	35.0 ± 9.7**	139.5 ± 199.3**	210.5 ± 249.3**	204.0 ± 254.8**
Control	370.5 ± 123.1	462.0 ± 85.0	500.0 ± 0.0	500.0 ± 0.0
Survival				
Hypothermia	10/10	8/10^#^	6/10^#^	6/10^#^
Control	8/10	2/10	0/10	0/10

**P < 0.01 hypothermic versus normothermic control with student's *t*-test.

^#^ *P* < 0.05 hypothermic versus normothermic control with Fisher's exact test.
